# Added Inspiratory Resistance Does Not Impair Cognitive Function and Mood State

**DOI:** 10.3390/ijerph20032743

**Published:** 2023-02-03

**Authors:** Yongsuk Seo

**Affiliations:** Sports AIX Graduate Program, Pohang University of Science and Technology, Pohang 37673, Republic of Korea; yseokss@postech.ac.kr

**Keywords:** inspiratory resistance, cognitive function, total mood disturbance, exercise

## Abstract

This study evaluated cognitive function and mood state with inspiratory resistance before and after maximal exercise in hypoxia. Nine healthy men (age = 25 ± 2 years) performed the Automated Neuropsychological Assessment Metrics—4th Edition (ANAM4) of the Stroop color–word test (SCWT) and total mood disturbance (TMD) before and after an incremental cycling exercise until volitional fatigue with four different inspiratory resistances (0, 1.5, 4.5, 7.5 cm H_2_O·L^−1^·s^−1^). There was no significant difference in the interference score of SCWT and TMD at normobaric, hypoxic conditions at four different inspiratory resistances. However, the interference score of SCWT was improved following maximal cycling exercise, whereas TMD was not improved. Inspiratory resistance did not have a deleterious effect on cognitive function and mood state in normobaric hypoxia after maximal cycling exercise. However, following maximal cycling exercise, cognitive function was improved.

## 1. Introduction

A broad array of occupations and workplaces require respiratory protective devices, also known as respirators, which are considered the first line of defense for protecting workers from inhaled chemical, biological, radiological, and nuclear substances when properly selected and applied. It is generally accepted that higher filtration rates will efficiently purify contaminated air, within the limits of the filtration material and fit of the respirator. However, higher filtration rates increase breathing resistance on negative-pressure respirators (such as a filtering facepiece respirator or elastomeric respirator). It has been shown that greater breathing resistance and work of breathing induce respiratory fatigue, which impairs physical work capacity, maximal work performance, and produces a perception of breathing discomfort and anxiety [1,2,3,4]. However, a previous study reported that low-intensity treadmill walking with a respirator did not impair cognitive tasks such as addition/subtraction, logical reasoning, or serial reaction, and did not impair mood state [1].

Physical activity, in moderate hypoxia, is common in occupational and military personnel. Hypoxia is defined as a lack of oxygen (O_2_) caused by a reduction in partial pressure of O_2_ in ambient air or in the O_2_ concentration of inspired air, which induce physiological and psychological alternations [5,6]. Hypoxia-induced cognitive impairments have been well documented and levels of arterial O_2_ saturation play a significant role in cognitive impairment. However, conflicting results have been reported, especially during moderate hypoxia, which describe a slowed reaction time at an altitude of 1500 m (17.6% O_2_) [7]. On the other hand, well-learned cognitive, vigilance, and perceptual–motor performance tasks can be maintained at an altitude of 2438 m (15.4% O_2_) [8,9]. Specifically, psychomotor performance, mental skills, reaction time, vigilance, memory, and logical reasoning have been reported to be impaired at an altitude of 3000 m and higher [10,11]. One review reported that acute exposure to altitude over 1500 m (720 torr) or breathing with less than 17.5% oxygen reduces maximal oxygen uptake (VO_2_ max) [12,13,14]. The reduction in VO_2_ max is minimal until about 1500 m, and linearly declines by 10% every 1000 m increase in altitude above 1500 m [15]. 

A recently published paper reported that inspiratory loading alters brain activity and deteriorates certain cognitive function, such as in the Paced Auditory Serial Addition Test (PASAT-A and B; calculation capacity, working memory, attention), the Trail Making Test (TMT-A, visuospatial exploration capacity; TMT-B, visuospatial exploration capacity, and attention) [16]. Inspiratory loading activates the respiratory-related cortical network and is associated with motor–cognitive interference [16,17,18]. 

Most occupational activities require submaximal effort and aerobic metabolism. However, there are still occupations that require physically demanding tasks (maximum or near-maximum effort) such as firefighters, first responders, and mining activities. Firefighters and first responders perform a variety level of aerobic and anaerobic activities while wearing respirators [19]. Anaerobic (high-intensity) activities produce and accumulate lactate in muscle and blood, which is considered a byproduct of anaerobic glycolysis [20,21].

Additionally, the concentration of cerebrospinal fluid lactate is highly related to cognitive function, human brain energy metabolism, and information interaction [22,23]. Review articles have reported that a moderate-intensity exercise below the lactate threshold or ranging from 25% to 70% maximal oxygen uptake (VO_2_ max), improves cognitive performance, with further increases in exercise intensity impairing cognitive performance [24,25]. Therefore, the effects of inspiratory resistance on cognitive function and mood state during higher exercise intensities in moderate hypoxic conditions are less clear. As such, the purpose of this study was to examine the effects of inspiratory resistance on cognitive function and mood state after maximal exercise. It was hypothesized that maximal exercise with added inspiratory resistance in moderate hypoxia would impair cognitive function and mood state. It was also hypothesized cognitive function impairment would be linear with the level of inspiratory resistance.

## 2. Material and Methods

The present study employed a single-blind, repeated measures design with a counterbalanced order. Each subject reported to the physiology laboratory on five separate occasions (one familiarization and four experimental trials), separated by at least three days. 

Experimental trials were conducted using a respiratory mask equipped with four custom-built inspiratory resistors (R) [(0, 1.5, 4.5, 7.5 cm H_2_O/L/s) at an airflow rate of 85 L/min] (model 7400 V-mask; Hans-Rudolph, Shawnee, KS). The Institutional Review Board of Kent State University (16-176) approved this study, and all subjects provided signed informed consent.

### 2.1. Participants

Nine healthy men (25 ± 2 years of age, height 181.4 ± 6.1 cm, weight = 92.5 ± 2.6 kg) participated in the current study. This sample size was determined based on a power analysis with an assumed power of 0.8 that nine participants would need to be recruited to reach statistical significance between four inspiratory resistances. 

No subjects had been exposed to normobaric hypoxia or hypobaric hypoxia within two months of the study. Subjects were screened via a health history questionnaire before participation and were excluded if they reported any history of cardiovascular disease, metabolic disorder, or respiratory disease, or were exposed to normobaric hypoxia or an altitude above 2500 m within two months prior to participation. Before participation, subjects were introduced to the normobaric hypoxia chamber (Colorado Altitude Training, Louisville, CO, USA) and were familiarized with the study protocol and instrumentation, including the cognitive function and mood state tests, at least three times. Subjects were instructed to abstain from alcohol, caffeine, and strenuous exercise for at least 24 h before each experimental trial. 

### 2.2. Experimental Procedure

On the days of experimental trials, subjects reported to the Exercise Physiology Laboratory at Kent State University following a 3 h self-reported fast intended to stabilize substrate utilization and reduce the risk of nausea during exercise in the hypoxic chamber. Subjects were initially equipped with a digit pulse-oximeter (Oxi-Go, Roslyn, NY, USA) for peripheral oxygen saturation measurement (SpO_2_), and a heart rate monitor (HR) (Polar RS800 CX, Polar Electro Oy, Kempele, Finland). Upon completion of instrumentation, subjects sat quietly on a chair for 10 min in ambient air, and baseline measurements of SpO_2_, HR, blood pressure (BP), and blood sample for peripheral blood lactate (LAC) (Nova Biomedical, Waltham, USA) were obtained. The Stroop color–word test (SCWT) and total mood disturbance (TMD) were assessed using an Automated Neuropsychological Assessment metrics—4th Edition (ANAM4) (Vista Life Sciences, Parker, CO, USA). After baseline, the subjects were moved into the normobaric hypoxia chamber and rested for 30 min while breathing through a silicone–rubber oro-nasal facemask that was outfitted with one of the aforementioned custom-built inspiratory resistors (R) [(0, 1.5, 4.5, 7.5 cm H_2_O/L/s) at an airflow rate of 85 L/min] (model 7400 V-mask; Hans-Rudolph, Shawnee, KS). At the end of the seated rest in hypoxia, SpO_2_, VO_2_, LAC, SCWT, and TMD results were obtained. 

**Figure 1:**
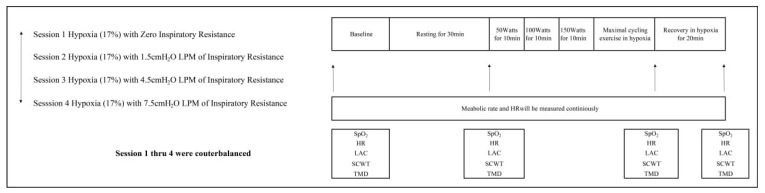
The experimental procedure and timeline. SpO_2_: peripheral oxygen saturation; HR: heart rate; LAC: blood lactate; SCWT: Stroop color–word test; TMD: total mood disturbance.

Upon the completion of tests during rest in hypoxia, subjects performed incremental cycling until volitional fatigue. The cycling exercise consists of three different work roads at 50, 100, and 100 watts for 10 min each, after which exercise intensity increased by 25 watts for every minute until volitional fatigue. The SpO_2_, VO_2_, LAC, SCWT, and TMD were obtained immediately after the cycling exercise, and then at 20 min of recovery in hypoxia (Figure 1). 

The SCWT assesses processing speed, selective attention, interference, and executive function. The SCWT consists of three 45 s tests. The first test involves pressing a corresponding key for each word (i.e., 1 for red, 2 for green, and 3 for blue). The next test requires pressing the corresponding key based on color. A series of colors (red, green, or blue) is presented on the screen. In the final test, a series of words (red, green, and blue) is presented in a color that does not match the name of the color displayed by the word. The subjects are required to press the response key assigned to color [26]. The mood state test assesses the transient mood state that consists of seven categories: anger, anxiety, depression, fatigue, happiness, restlessness, and vigor. Subjects were asked to rate 42 presented emotional words between 0 (Not at all) and 6 (Very much). Total mood disturbance (TMD) was calculated as: negative mood (sum of anger, anxiety, depression, fatigue, and restlessness)—positive mood (sum of vigor and happiness). A higher TMD score indicates a poorer mood state. 

### 2.3. Statistical Analyses

Using a statistical software package (SPSS v. 19.0, IBM, Armonk, NY, USA), two-way (four conditions by four-time points) repeated measures analysis of variance (ANOVA) was utilized to ascertain the effect of inspiratory resistance on dependent variables. When ANOVA indicated a significant main effect and interaction, a post hoc pair-wise comparison with the least significant difference was conducted. Statistical significance was set at *p* ≤ 0.05; all data are presented as the mean ± standard deviation (SD). 

## 3. Results

Baseline measurements of blood LAC (F = 0.769, *p* = 0.523), SpO_2_ (F = 0.388, *p* = 0.762), interference score for SCWT (F = 1.183, *p* = 0.337), and TMD (F = 2.158, *p* = 0.119) did not differ between inspiratory resistances. 

**Figure 2:**
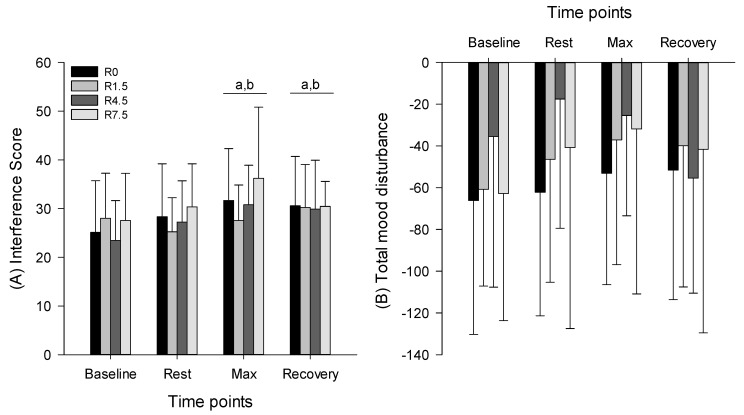
(**A**) Interfere score for the Stroop color–word test and (**B**) total mood disturbance during rest and maximal cycling exercise. ^a^ *p* ≤ 0.05 vs. baseline; ^b^ *p* ≤ 0.05 vs. resting in hypoxia.

The interference score for SCWT indicated the main effect for time (F = 5.214, *p* = 0.006, power = 0.878), but no main effect for condition (F = 1.859, *p* = 0.163, power = 0.420) and no interaction (F = 1.263, *p* = 0.272, power = 0.572). The interference score was improved after maximal exercise compared with baseline (*p* = 0.007) and resting in hypoxia (*p* = 0.041). During recovery, the interference score was also improved compared with baseline (*p* = 0.045) and resting in hypoxia (*p* = 0.015) (Figure 2A).

The TMD indicated no main effect for time (F = 1.413, *p* = 0.263, power = 0.326), no main effect for condition (F = 1.117, *p* = 0.362, power = 0.263), and no interaction (F = 1.407, *p* = 0.201, power = 0.629) (Figure 2B).

Measurements of LAC, HR, and SpO_2_ between conditions and time are presented in Table 1. LAC indicated a main effect for time (F = 71.295, *p* ≤ 0.001, power = 1.0) and interaction (F = 2.116, *p* = 0.039, power = 0.838), but no main effect for condition (F = 1.702, *p* = 0.193, power = 0.388). Post hoc pair-wise comparison revealed that LAC was significantly lower during R7.5 than R1.5 (*p* = 0.046). As expected, LAC was significantly increased following maximal exercise compared with the baseline (*p* ≤ 0.001) and resting in hypoxia (*p* = 0.002). Furthermore, LAC was significantly decreased during recovery compared with maximal exercise (*p* ≤ 0.001). 

HR indicated no main effect for condition (F = 2.590, *p* = 0.076, power = 0.562), a main effect for time (F = 104.463, *p* ≤ 0.001, power = 1.0), and no interaction (F = 1.346, *p* = 0.229, power = 0.606). HR was significantly increased following maximal exercise compared with baseline (*p* ≤ 0.001) and resting in hypoxia (*p* ≤ 0.001). HR was significantly decreased during recovery compared with maximal exercise (*p* ≤ 0.001).

SpO_2_ indicated no main effect for condition (F = 0.986, *p* = 0.416), a main effect for time (F = 19.115, *p* ≤ 0.001), or no interaction (F = 1.053, *p* = 0.408). SpO_2_ was significantly decreased during rest, maximal exercise, and recovery compared with baseline (all *p* ≤ 0.001). 

The maximal power output was significantly lower in R7.5 (*p* = 0.016) and R4.5 (*p* = 0.05) than R0. Furthermore, power output was significantly lower in R7.5 (*p* = 0.035) than R1.5

## 4. Discussion

This study examined the cognitive function and mood state during maximal exercise with added inspiratory resistance at normobaric hypoxic conditions. It was hypothesized that maximal exercise with added inspiratory resistance at normobaric hypoxic conditions would impair cognitive function and mood state. The main findings of the study were (1) there was no significant impairment in cognitive function at normobaric, hypoxic conditions at various inspiratory resistances, whereas additional inspiratory resistance decreased the maximal power output with increasing inspiratory resistances; (2) total mood disturbance was not significantly affected by inspiratory resistance. It has previously been reported that a 15% reduction in arterial oxygen saturation results in diminished concentration capacity and impaired muscular coordination [27]. In the current study, oxygen saturation was maintained above 90% under all study conditions; thus, oxygen delivery to the brain was sufficient for normal brain function. Many previous studies have reported differing findings on the impact of hypoxia on cognition, with some reporting impairment and others noting no impact [28]. For example, at an altitude 37,000 feet on pressurized commercial airliners, the oxygen saturation is essentially the same as in the current study (17%) and healthy passengers are at no significant health risk [29]. At higher breathing resistance levels, oxygenation is maintained by an increase in the respiratory duty cycle (the proportion of time spent in the inspiratory phase) [30] accompanied by a decrease in the respiratory rate [31]. It is also likely that ventilation was sufficient to ensure that there was not a significant increase in CO_2_ retention, as this, too, would lead to cognitive impairment. The lower heart rate noted with R7.5 at maximal exercise may be related to the decreased intrathoracic pressures generated by the respiratory muscles to overcome the increased resistance of R7.5, resulting in increased stroke volume and cardiac output [31]. The increased cardiac output would have also contributed to improved blood flow to the lower extremity muscles and the respiratory muscles, thereby leading to the lower LAC level noted with R7.5 at Max (Table 1). Recent studies suggest that blood lactate metabolism may contribute to the improvements in attentional or executive performance [32]. An increase in blood lactate after high-intensity exercise could be associated with improved attentional or executive performance [33,34,35]. 

Coco et al. [36] reported that lactate is an important energy substrate in astrocytes, and plays a protective or modulatory role at the level of primary cortical areas (such as M1, V1, or S1) [36]. Similarly, the lack of a significant difference in mood between levels of inspiratory resistance indicates that cerebral blood flow was not significantly impaired, given that lower cerebral blood flow can have a negative impact on mood [37]. Interestingly, the present study did not detect a deleterious effect of maximal exercise on cognitive function and mood state, although others reported impaired cognitive function and mood state following maximal exercise without any breathing resistance [25,38]. One possible explanation is that inspiratory resistance stimulates sympathetic tone and induces arousal. A previous study reported that respiratory-effort-related arousals have been shown to increase cardiac sympathetic tone by the low-frequency power of heart rate variability [39]. This activation of sympathetic tone may prevent impairments of cognitive function. 

Furthermore, a recent review paper reported that cognitive function after high-intensity exercise may depend on the timing of the cognitive task, type of cognitive task, and exercise mode/duration [40]. In a previous review study, improvements, impairments, and no changes have all been observed within 5 min of the high-intensity exercise being completed [40]. However, cognitive performance was not impaired when cognitive tasks were performed 6 min after exercise [40]. 

Although this is the first study to examine the levels of inspiratory resistance and maximal exercise on cognitive function and mood state, some study limitations could be identified. First, the present study recruited a small sample of young, healthy men, which may have limited the ability to detect the change in cognitive function, as indicated by the low statistical power. It is necessary to evaluate a broader range of populations such as older adults, women, or ethnicity to generalize the results. Therefore, the present study needs to be interpreted with caution.

Furthermore, we were unable to determine the influence of inspiratory resistance on cognitive function. A larger and more diverse sample, such as older adults or women, may provide clear results and enhance the sensitivity of the statistical differences because the number of women and older adults in the workforce is growing [41]. Second, it might be beneficial to measure arousal and sympathetic tone. The sympathetic tone or arousal were not obtained in relation to cognitive function, because this function is not available in our laboratory. 

## 5. Conclusions

The present study demonstrated that inspiratory resistance did not impair the interference score for SCWT during rest in hypoxia, while the interference score for SCWT was improved after maximal exercise and recovery. Inspiratory resistance in hypoxia did not induce deleterious effects on mood state, despite higher blood lactate levels.

## Figures and Tables

**Table 1:** Peripheral oxygen saturation (SpO_2_), heart rate (HR), lactate (LAC), and power output during baseline, resting, maximal exercise, and recovery at four different inspiratory resistance levels.

		Base	Resting	Max	Rec
SpO_2_ (%)	R0	97.9 ± 0.8	93.3 ± 3.3 ^a^	92.8 ± 3.2 ^a^	93.3 ± 2.6 ^a^
	R1.5	98.0 ± 0.9	92.8 ± 3.3 ^a^	95.1 ± 2.3 ^a^	95.0 ± 2.0 ^a^
	R4.5	97.8 ± 0.7	94.3 ± 3.4 ^a^	92.8 ± 2.6 ^a^	94.2 ± 2.2 ^a^
	R7.5	97.7 ± 0.5	93.8 ± 2.8 ^a^	93.3 ± 2.6 ^a^	94.2 ± 3.0 ^a^
HR (beats/min)	R0	69.1 ± 8.2	68.7 ± 7.5	154.3 ± 21.4 ^a,b,c^	91.7 ± 8.4 ^a,b,c^
	R1.5	66.1 ± 6.8	67.1 ± 7.4	143.2 ± 28.1 ^a,b,c^	86.9 ± 5.4 ^a,b,c^
	R4.5	63.1 ± 7.7	66.0 ± 10.3	141.1 ± 23.5 ^a,b,c^	88.9 ± 6.5 ^a,b,c^
	R7.5	66.1 ± 8.7	67.9 ± 9.4	131.1 ± 26.0 ^a,b,c^	87.3 ± 8.0 ^a,b,c^
LAC (mmol/L)	R0	1.5 ± 0.5	1.3 ± 0.5	10.8 ± 1.7 ^a,b,c^	6.5 ± 3.2 ^a,b,c^
	R1.5	1.6 ± 1.2	1.7 ± 1.2	10.9 ± 3.6 ^a,b,c^	6.3 ± 3.4 ^a,b,c^
	R4.5	1.4 ± 0.5	2.2 ± 1.5	10.2 ± 2.2 ^a,b,c^	6.4 ± 3.5 ^a,b,c^
	R7.5	1.9 ± 0.8	2.0 ± 1.1	9.1 ± 2.5 ^a,b,c^	4.5 ± 3.0 ^a,b,c^
Power output (Watts)	R0	-	-	272.2 ± 44.1	-
	R1.5	-	-	263.9 ± 41.7	-
	R4.5	-	-	255.6 ± 34.9 *	-
	R7.5	-	-	241.7 ± 50.0 *^,#^	-

Values are mean ± SD. R0: zero inspiratory resistance; R1.5: 1.5 cmH_2_O LPM of inspiratory resistance; R4.5: 4.5 cmH_2_O LPM of inspiratory resistance; R7.5: 7.5 cmH_2_O LPM of inspiratory resistance. Base: baseline; Resting: resting in hypoxia; Max: maximal cycling exercise in hypoxia; Rec: recovery in hypoxia; HR: heart rate; LAC: blood lactate ^a^ *p* ≤ 0.05 vs. baseline; ^b^ *p* ≤ 0.05 vs. resting; ^c^ *p* ≤ 0.05 vs. max. * *p* ≤ 0.05 vs. R0; ^#^ *p* ≤ 0.05 vs. R1.5.

## Data Availability

The data presented in this study are available on request from the corresponding author. The data are not publicly available due to the protection of personal information.
